# Arboviruses circulation in Guinea: Overview and perspectives for public health

**DOI:** 10.1371/journal.pntd.0013904

**Published:** 2026-01-09

**Authors:** Salifou Talassone Bangoura, Alpha-Kabinet Keita, Sidikiba Sidibé, Saidouba Cherif Camara, Maladho Diaby, Kadio Jean-Jacques Olivier Kadio, Abdoul Karim Soumah, Haby Diallo, Alexandre Delamou, Eric Delaporte, Alioune Camara, Michèle Ottmann, Nagham Khanafer, Abdoulaye Touré

**Affiliations:** 1 Centre de Recherche et de Formation en Infectiologie de Guinée (CERFIG), Gamal Abdel Nasser University, Conakry, Guinea; 2 Department of Public Health, Gamal Abdel Nasser University, Conakry, Guinea; 3 Recherches Translationnelles sur le VIH et les Maladies Infectieuses (TransVIHMI), Institut de Recherche pour le Développement, University of Montpellier, Montpellier, France; 4 African Centre of Excellence in the Prevention and Control of Communicable Diseases (CEA-PCMT), Faculty of Sciences and Health Techniques, Gamal Abdel Nasser University, Conakry, Guinea; 5 Virpath Team, Centre International de Recherche en Infectiologie, Inserm U1111, CNRS UMR5308, ENS de Lyon, Lyon 1 University, Lyon, France; 6 Infection Control Unit, Hôpital Edouard Herriot, Hospices Civils de Lyon (HCL), Lyon, France; 7 PHE3ID Team, Centre International de Recherche en Infectiologie (CIRI), Inserm U1111, CNRS UMR5308, ENS de Lyon, Lyon 1 University, Lyon, France; Monash University, AUSTRALIA

## Abstract

**Background:**

Arboviruses constitute a major global public health concern, particularly in countries with fragile healthcare systems. However, data on arbovirus circulation remain fragmented and under-analysed. This study aimed to document the historical and current circulation of arboviruses in Guinea and propose public health strategies to enhance surveillance, prevention, and control.

**Methodology/principal findings:**

We compiled reports of arbovirus-related human epidemics in Guinea from 2000 to 2024, drawing information from Guinea’s weekly epidemiological bulletins, as well as from the World Health Organization’s Weekly Bulletin on Outbreaks and Other Emergencies, the Africa CDC’s Weekly Event-Based Surveillance Report, and the Annual Global Yellow Fever Update reports. In addition, a literature review was conducted to identify studies on arboviruses involving arboviruses in humans, vectors, and animals. Between 2000 and 2024, multiple and simultaneous arboviral outbreaks were reported by surveillance systems, involving three arboviruses: yellow fever virus (YFV), dengue virus (DENV), and chikungunya virus (CHIKV). YFV outbreaks were the most recurrent, with 947 confirmed cases and 284 deaths, corresponding to a case fatality rate of 30%. Moreover, 17 studies documented the circulation of several arboviruses in humans, vectors, and animals, including DENV, YFV, CHIKV, Zika virus (ZIKV), and West Nile (WNV). Novel viruses such as Kindia virus, Forécariah virus, and Kolente virus have also been identified. Serological evidence in human reported IgM seroprevalence ranging from 1.2% to 14.7% for ZIKV, 4.3% to 12.2% for YFV, and 0.6% to 2.1% for DENV. Additionally, other studies were also reported IgM seroprevalence of 23.4% for WNV, 17.0% for CHIKV, and 10.6% for TAHV. Concerning IgG serology, the studies reported seroprevalences ranging from 12.9% to 51.7% for CHIKV, 27.0% to 34.0% for WNV, 11.8% to 25.2% for DENV, and 2.2% to 3.0% for CCHFV. Furthermore, IgG seroprevalences of 28.5% for YFV and 5.5% for BATV were reported.

**Conclusions:**

This study highlights the circulation of several arboviruses in Guinea across human, vector animal populations. The results suggest that public health efforts should focus on community engagement, strengthened entomological and epidemiological surveillance, and multisectoral and international collaboration, to anticipate, prevent, and control future arbovirus-related epidemics.

## Author summary

Arboviruses represent an increasing global public health threat, particularly in countries with fragile healthcare systems, where their burden remains largely underestimated. In Guinea, data on arbovirus circulation remain limited. This study analysed information from epidemiological surveillance systems and scientific literature to provide a historical and current overview of arboviruses circulating in the country. Between 2000 and 2024, several outbreaks involving yellow fever, dengue, and chikungunya were recorded. Yellow fever was the most recurrent, with a case fatality rate of 30%. However, serological evidence indicates the circulation of various arboviruses in humans, with high IgM seroprevalence reported particularly for Zika, West Nile, and Tahyna viruses. In addition, emerging viruses such as Kindia, Forécariah, and Kolente were identified, highlighting the diversity of arboviruses in Guinea. These findings highlight the urgent need to strengthen entomological and epidemiological surveillance, engage communities, and promote multisectoral coordination to prevent the future arboviral epidemics.

## Introduction

Arbovirus-related diseases represent a major global concern, placing significant pressure on healthcare systems and impeding both local and global economic and social development [1,2]. The frequency of arboviral outbreaks, particularly those transmitted by mosquitoes, has been increasing significantly worldwide [3]. It is estimated that nearly half of the global population is at risk of contracting dengue, with an estimated 100–400 million cases occurring annually [4]. Africa accounts for 16% of all dengue cases, making it the second most affected region globally [5]. A recent study reported that, in 2023 alone, 29 arboviral outbreaks occurred across 25 African countries [6]. Furthermore, a meta-analysis of seroprevalence data over the past two decades suggests a wider circulation of various arboviruses in sub-Saharan Africa, although their transmission dynamics remain poorly understood [7].

A recent assessment of health system capacities in the 47 countries of the World Health Organization (WHO) African Region revealed significant gaps in arbovirus surveillance in humans, vectors, and animals, hampering early outbreak detection [8]. Key challenges included limited virological and entomological surveillance, insufficient control of vectors such as *Aedes* mosquitoes, and a lack of community awareness and engagement in arbovirus disease prevention and control activities [8].

In Guinea, several arboviral diseases, such as dengue, yellow fever, Rift Valley fever, and Crimean-Congo hemorrhagic fever, are classified among the priority zoonoses under surveillance [9]. In response to recurrent outbreaks, notably those of Ebola virus disease, COVID-19, measles, Lassa fever, and Marburg virus, substantial investments have been made to strengthen the epidemiological surveillance system for epidemic-prone diseases across human and animal populations [10]. However, further efforts are required to enhance early detection and rapid response capabilities, and these remain a priority for many research institutions.

Several factors suggest the frequent circulation of arboviruses in Guinea: the presence of competent mosquito vectors for numerous arboviruses; a high burden of other mosquito-borne diseases such as malaria; and the recurrent documentation of yellow fever outbreaks. Additionally, Guinea’s geographical proximity to countries with frequent arboviral outbreaks further supports this hypothesis, as increased human mobility and cross-border movement facilitate the introduction and spread of arboviruses and their vectors [11–13]. Other factors, such as the country’s climatic conditions (seasonal rainfall and rising temperatures) [14], rapid urbanization, inadequate infrastructure [15,16], and poor water and waste management, contribute to vector proliferation and facilitate the circulation of arboviruses [17].

However, arboviral infections are not routinely included in the diagnostic practices of healthcare facilities in Guinea, which may lead to underdetection until large-scale outbreaks occur. The limited understanding of arboviral epidemiology in the region hinders the analysis of transmission dynamics and impairs the ability to prevent and anticipate future outbreaks. Such information is essential to inform the design of effective surveillance and intervention strategies aimed at reducing the overall burden of morbidity and mortality associated with these diseases.

The objective of this study was to document the historical and current circulation of arboviruses in Guinea and to propose public health perspectives aimed to strengthen surveillance, prevention, and control strategies.

## Methods

### Ethics statement

All data used in this study were obtained from publicly available and open-access sources. Therefore, ethical approval was not required. Nonetheless, formal authorization for the use of Guinea’s surveillance data was obtained from the National Health Safety Agency of Guinea.

### Study area

The Republic of Guinea is located in southwestern West Africa, and covers an area of 245,857 km², with an estimated population of 14,363,931 in 2025 [18]. It shares borders with Guinea-Bissau to the northwest, Senegal and Mali to the north, Côte d’Ivoire and Mali to the east, Liberia and Sierra Leone to the south, and the Atlantic Ocean to the west.

It is subdivided into eight administrative regions: Conakry (the capital), Boké, Faranah, Kankan, Kindia, Labé, Mamou, and N’zérékoré [19], as well as 38 health districts.

Climatic conditions vary across regions. Lower Guinea (Boké and Kindia) receives high annual rainfall ranging from 3,000–4,000 mm. Middle Guinea (Labé and Mamou) receives between 1,500 mm and 2,000 mm, while Upper Guinea (Faranah and Kankan) has lower annual rainfall levels of 1,000 mm to 1,500 mm. The Forested region (N’zérékoré) receives between 2,000 mm and 3,000 mm of rainfall annually. Malaria is endemic throughout the country, with stable transmission and seasonal peaks from June to September [20].

### Organization of epidemiological surveillance in guinea

In Guinea, surveillance, investigation, and response to epidemic-prone infectious diseases are coordinated by the Ministry of Health (MoH) [10]. Before the emergence of Ebola virus disease (EVD), each health facility directly contacted the available laboratories to analyse samples from patients suspected of infectious diseases. Following the 2014–2016 EVD outbreak, national diagnostic capacities were strengthened through the establishment of specialized research laboratories.

The healthcare facilities are responsible for identifying and reporting suspected arboviral disease cases, in accordance with the technical Guidelines for Integrated Disease Surveillance and Response in the African Region (IDSR) in the African Region [21]. For each suspected case, a notification form is completed and a venous blood sample is collected. These samples are sent to designated laboratories, for molecular or serological (IgM) testing. In the event of laboratory confirmation, the MoH initiates an in-depth investigation to collect the data necessary to confirm the outbreak, while simultaneously implementing initial response measures. Subsequently, samples are sent to the Pasteur Institute in Dakar, such as in the case of yellow fever, for confirmation prior to notification to the WHO.

### Data sources and research strategy

We compiled available reports of arbovirus-related human epidemics in Guinea up to the end of 2024, drawing Guinea’s weekly epidemiological bulletins, as well as from the World Health Organization’s Weekly Bulletin on Outbreaks and Other Emergencies, the Africa CDC’s Weekly Event-Based Surveillance Report, and the Annual Global Yellow Fever Update reports. The dataset included the health district where the outbreak occurred, the type of arbovirus, the date the first cases were reported, the number of confirmed cases, the number of deaths, and the corresponding data sources.

In addition, a literature search was conducted on PubMed, Web of Science, Scopus, and ResearchGate to identify studies reporting arbovirus circulation in Guinea in humans, vectors (mosquitoes and ticks), or animals. Search terms included: [“arbovirus” OR “arboviruses” OR “arthropod-borne virus” OR “dengue” OR “chikungunya” OR “yellow fever” OR “zika” OR “West Nile virus” OR “Rift Valley fever virus” OR “Crimean-Congo hemorrhagic fever” OR “mosquito-borne disease”] AND [“Guinea”]. Relevant reference lists from selected articles were also screened. No restrictions were applied regarding publication date (up to the end of 2024) or language.

### Data analysis

Data were compiled in Microsoft Excel and analysed using RStudio software (version 4.4.2). Duplicate entries were identified and removed based on notification dates and case descriptions.

Descriptive statistics were applied to summarize the data. Geographic distribution of cases was visualized using QGIS software (version 3.36.1), with administrative boundaries sourced from the Global Administrative Areas GADM) database (https://www.gadm.org/), licensed under terms specified at: https://www.gadm.org/license.html.

## Results

### Arboviral outbreak trends and geographical distribution

Between 2000 and 2024, multiple and simultaneous arboviral outbreaks were reported by surveillance systems, with recurrent YFV outbreaks. In total, 947 confirmed YFV cases and 284 associated deaths were recorded, corresponding to a case fatality rate (CFR) of 30%. The largest outbreak occurred between September 2000 and January 2001, affecting 17 health districts, with 833 cases and 246 deaths (CFR: 29.5%) (Table 1). Moreover, two confirmed cases of DENV, including one death, and one case of CHIKV were reported between 2023 and 2024 (Table 1).

**Table 1:** Distribution of arbovirus-related cases and deaths in Guinea, 2000–2024.

Arbovirus	Years	Health district	Confirmed cases	Deaths	CFR (%)^a^
Yellow fever virus	2000-2001	17 health districts	833	246	29.5
	2005	Ratoma, Nzérékore, Dalaba, Telimélé, Kindia, Koundara, Matoto, Faranah, Koubia, Boké	74	33	44.6
	2008	Faranah	2	0	0
	2010	Mandiana	4	0	0
	2016	*ND*	5	*ND*	–
	2018	Mandiana	1	0	0
	2019	Boké and Koubia	5	2	40.0
	2020	Koundara, Beyla, Koubia, Kouroussa	11	Unknown^b^	–
	2022	Dabola	1	1	100
	2023	Dinguiraye, Koundara, Guéckédou	3	1	33.3
	2024	Koundara, Lelouma, Gaoul	8	1	12.5
	**Total**		**947**	**284**	**30.0**
Dengue virus	2023	Ratoma	1	1	100
	2024	Matam	1	0	0
	**Total**		**2**	**1**	**50.0**
Chikungunya virus	2024	Dalaba	1	0	0
	**Total**		**1**	**0**	**0**

^a^ CFR: case-fatality rate in confirmed cases.

^b^ Unknown, but 14 deaths among suspected cases.

ND: Not defined.

**Fig 1:**
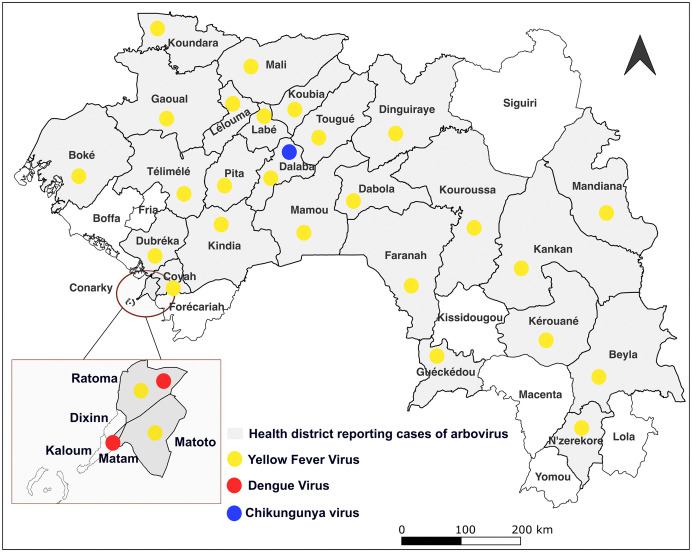
Map of Guinea showing the health districts that reported at least one arbovirus outbreak by the surveillance system between 2000 and 2024. Map created with QGIS software (source of administrative boudaries map layer:https://www.gadm.org/; Link to the GADM license: https://www.gadm.org/license.html).

Fig 1 shows that YFV outbreaks occurred in nearly all health districts, except for a few. In contrast, dengue outbreaks were recorded only in the capital city, Conakry, specifically in the districts of Ratoma and Matam. Moreover, one confirmed case of chikungunya was reported in the health district of Dalaba (Fig 1).

### Research on arboviruses in Guinea

**Fig 2:**
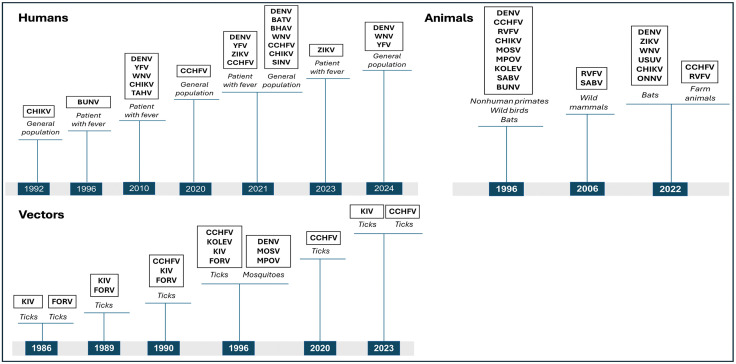
Timeline of arbovirus studies conducted in Guinea in humans, vectors, and animals (up to the end of 2024). BATV: Batai virus; BHAV: Bhanja virus; BUNV: Bunyamwera virus; CCHFV: Crimean-Congo hemorrhagic fever virus; CHIKV: Chikungunya virus; DENV: Dengue virus; FORV: Forecariah virus; KIV: Kindia virus; KOLEV: Kolente virus; MOSV: Mossuril virus; MPOV: M’Poko virus; ONNV: O’nyong-nyong virus; RVFV: Rift Valley fever virus); SABV: Saboya virus; SINV: Sindbis virus; TAHV: Tahyna virus; USUV: Usutu virus; WNV: West Nile virus; YFV: Yellow fever virus; ZIKV: Zika virus.

A total of 17 studies were identified from electronic databases. These studies conducted on human (8 studies), mosquitoes (1 study), ticks (7 studies), bats (2 studies), nonhuman primates (1 study), wild mammals (1 study), wild birds (1 study), and farm animals (1 study) (Fig 2) (S1 Table).

**Fig 3:**
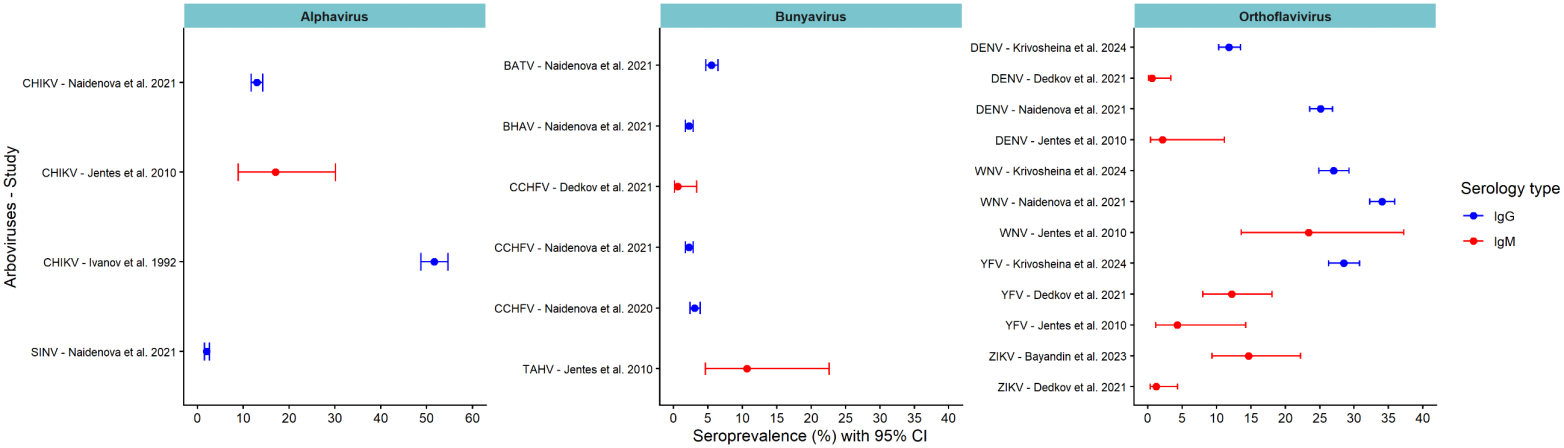
Seroprevalence of human arboviruses by genus: evidence from studies conducted in Guinea (up to the end of 2024). BATV: Batai virus; BHAV: Bhanja virus; BUNV: Bunyamwera virus; CCHFV: Crimean-Congo hemorrhagic fever virus; CHIKV: Chikungunya virus; DENV: Dengue virus; SINV: Sindbis virus; TAHV: Tahyna virus; WNV: West Nile virus; YFV: Yellow fever virus; ZIKV: Zika virus.

Serological data from patients with fever showed IgM seroprevalences ranging from 1.2% to 14.7% for ZIKV, 4.3% to 12.2% for YFV, and 0.6% to 2.1% for DENV. Additionally, other studies also reported IgM seroprevalence of 23.4% for WNV, 17.0% for CHIKV, and 10.6% for TAHV. Concerning IgG serology, studies in the general population reported seroprevalences ranging from 12.9% to 51.7% for CHIKV, 27.0% to 34.0% for WNV, 11.8% to 25.2% for DENV, and 2.2% to 3.0% for CCHFV. Moreover, IgG seroprevalences of 28.5% for YFV and 5.5% for BATV were reported (Fig 3).

Studies conducted on vectors have isolated several arboviruses such as DENV, Mossuril virus (MOSV) and M’Poko virus (MPOV) from mosquitoes. In addition, CCHFV has been isolated from ticks as well as novel viruses including Kolente virus (KOLEV), Kindia virus (KIV), and Forécariah virus (FORV) (Fig 2) (S1 Table).

Furthermore, studies conducted on animals have isolated DENV, RVFV and CHIKV in non-human primates, ZIKV, WNV, Usutu virus (USUV) and CHIKV in bats, and Saboya virus (SABV) in wild mammals and birds (Fig 2) (S1 Table).

## Discussion

Describing the circulation of arboviruses in Guinea is crucial for assessing the public health impact, particularly in a context marked by overlapping viral infections with similar clinical manifestations. This knowledge is essential for guiding public health strategies and strengthening preparedness and response capacities to future outbreaks.

The results indicate a persistent occurrence of yellow fever outbreaks affecting several health districts in Guinea, leading to increased morbidity and mortality. This situation may be attributable to several factors, most notably the low vaccination coverage, estimated at 40% in 2023 [22–24], despite Guinea’s YFV control strategy, implemented for decades, primarily relies on vaccination for infants from nine months and travellers to endemic areas, supplemented by mass vaccination campaigns [25]. This level is far below the 80% threshold required to achieve herd immunity against yellow fever, leaving a substantial proportion of the population susceptible to infection and thereby sustaining viral transmission [26]. Similar patterns have been observed in several other African countries, where most yellow fever cases occur among unvaccinated populations living in high-risk areas [27]. Africa accounts for 90% of the global yellow fever burden, and a large part of the continent is classified by the WHO as a high-risk area for yellow fever transmission [28]. From 1 January 2021–7 December 2022, 203 confirmed cases and 252 probable cases, including 40 deaths (case fatality rate of 9%), were reported across 13 countries in the WHO African Region [29]. In 2023, nine countries reported 104 confirmed yellow fever cases, including 39 deaths [6]. Furthermore, a modelling study in 2013 estimated that the yellow fever burden in Africa ranged from 84,000–170,000 severe cases and from 29,000–60,000 deaths [30]. Although the epidemiology of yellow fever is complex, involving both wild and domestic vectors in human-to-human transmission, the risk of outbreaks can be substantially reduced through improved vaccination coverage. The 17D yellow fever vaccine is safe, effective, affordable, and readily available, and a single dose provides long-lasting immunity and lifelong protection [28].

Beyond the YFV epidemics detected by the surveillance system, several studies conducted in Guinea have documented the circulation of multiple arboviruses in humans, including WNV, CHIKV, CCHFV, BUNV, and TAHV. A recent study among febrile patients reported an anti-ZIKV IgM seroprevalence of 14.7%, along with a PCR-confirmed case in a pregnant woman. This finding suggests recent or active virus transmission within the population, in a context of limited diagnostic capacity. Given the major public health consequences of ZIKV transmission, including neurological complications in adults and congenital malformations, such as microcephaly, in newborns exposed in utero [31], it is crucial to improve the understanding of ZIKV circulation dynamics within communities, identify factors that facilitate its transmission, and strengthen surveillance capacities to enable early detection of infections and guide targeted prevention and control interventions. This observation fits within an epidemiological context in which the circulation of the ZIKV virus has been documented in several neighbouring countries [32]. Since 2015, viral circulation episodes have been reported in Angola, Guinea-Bissau, and Cape Verde [33–35]. More recently, in 2023, outbreaks were confirmed in Mali and Senegal, totalling 24 reported cases without deaths [6]. Furthermore, several serological studies conducted in Africa have detected anti-ZIKV IgM in humans, indicating recent infections [36–39]. All of these observations highlight the need to strengthen epidemiological and virological surveillance in the subregion in order to anticipate and prevent potential outbreaks.

This study demonstrates that several novel arboviruses, namely KINV, FORV, and KOLEV, were identified in Guinea during the 1980s from ticks, and that their clinical and pathogenic characteristics, as well as their potential impact on public health, remain currently undetermined. FORV, isolated from *Boophilus geigyi* in the Forécariah prefecture, belongs to the Bhanja antigenic group; historical serological surveys indicate human exposure in endemic areas, although no clinical manifestations have been formally documented [40,41]. KINV comprises both historical isolates associated with the Palyam group and more recent isolates identified as “Kindia tick virus (KITV),” a segmented flavi-like virus related to Jingmenviruses, whose complete genome has been characterized recently but with no confirmed human disease association to date [42–45]. KOLEV, a highly divergent rhabdovirus isolated from ticks and a bat, encodes the five canonical rhabdoviral structural proteins and induces cytopathic effects in cell culture as well as neuro-pathogenicity in neonatal mice following intracranial inoculation, suggesting experimental neurotropism, although no human cases have been reported [46]. The absence of documented human clinical cases may reflect either genuinely low virulence in humans or a substantial lack of surveillance and diagnostic capacity, thereby limiting accurate assessment of these viruses’ true geographic distribution and pathogenic potential. In the context of an increasing emergence of novel arboviruses, KINV, FORV and KOLEV represent understudied but potentially relevant agents that warrant comprehensive investigations into their viral ecology, epidemiology, genomic evolution and pathogenesis to clarify their possible implications for public health.

The results of this study reveal a considerable discrepancy between officially reported cases and the reality of viral circulation, suggesting a major underestimation of the real burden of arboviruses in Guinea. This situation raises more general questions about the capacity of the national surveillance to effectively detect and respond to arbovirus outbreaks. Although Guinea has made efforts to strengthen disease surveillance, these remain insufficient for rapid case detection and timely epidemic response. One of the major challenges lies in diagnosing arboviral diseases due to frequent asymptomatic infections and the overlap of their clinical symptoms with other endemic diseases, particularly malaria. This makes clinical diagnosis especially difficult in health districts with limited virological laboratory capacity. In practice, arboviruses are often considered only if the malaria test is negative. Consequently, when a malaria test is positive, clinicians may not pursue further differential diagnosis. Thus, in the absence of confirmatory testing, many healthcare professionals frequently rely on a presumptive diagnosis of malaria for febrile illnesses. Moreover, the limited knowledge of medical staff about arboviruses represents a challenge for the early detection of cases and the surveillance of arboviruses. A recent assessment in Conakry revealed that only 1% of healthcare workers had good knowledge of arboviruses [47]. They are more likely to detect suspected cases of yellow fever than other arboviruses due to the yellowing of the eyes. It is therefore essential to inform and raise awareness among healthcare providers regarding the diagnosis, management, and prevention of arboviral diseases, as well as best practices and epidemiological surveillance. Such an approach would improve the early detection of outbreaks and effective response.

The results of this study reinforce the need for integrated surveillance systems that combine epidemiological, entomological and genomic approaches to better understand arbovirus dynamics. Additional research on the effects of environmental and climatic variables on vector ecology and transmission is also essential for anticipating outbreaks risks. The limitations of this study include the heterogeneity of surveillance reporting across health districts, which may lead to underreporting.

The absence of granular data also prevented analysis of spatial variations in transmission, limiting the ability to identify high-risk areas and tailor control efforts accordingly.

### Public health perspectives for arbovirus control in Guinea

**Fig 4:**
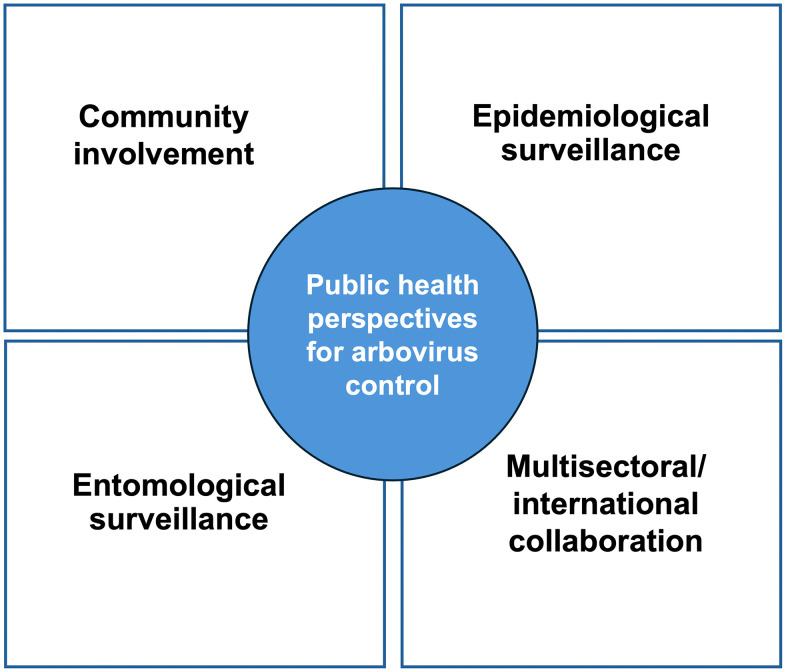
Public health perspectives for arboviruses control in Guinea.

Arbovirus control in Guinea requires an integrated approach that incorporates multiple public health strategies. Community engagement, entomological and epidemiological surveillance, and multisectoral and international collaboration are essential (Fig 4).

a) **Community engagement**

The mobilisation and engagement of local communities are key to infection prevention and vector control [48,49]. In the Guinean context, where health system resources remain limited and community-based interventions are essential, this approach requires the active involvement of local actors, such as religious leaders, elders, and youth and women’s organizations, to carry out awareness-raising and health education activities tailored to the communities’ sociocultural realities. Through simple messages and educational materials addressing environmental hygiene, waste management, and the individual and collective prevention of arboviral infections, these actors will help strengthen the adoption of good practices by the population and sustainably influence behaviors related to arbovirus transmission. Successful community-based initiatives in other contexts have shown significant reductions in vector density and disease transmission [50–54].

b) **Entomological surveillance**

Vector surveillance is critical for understanding the spatial and temporal dynamics of arbovirus transmission [55–57]. However, Guinea’s entomological capacity remains limited. Investments in mosquito surveillance have been low and primarily focused on malaria control, leaving surveillance of arbovirus vectors largely inadequate. To address these challenges, it is crucial to establish a vector surveillance program. This requires the development of a national strategic plan aligned with the 2017–2030 Global Vector Control Response [58], as well as the allocation of resources necessary to strengthen the country’s technical and operational capacities (e.g., insectariums labs, field stations, etc.). Additionally, continuous training of entomologists and laboratory technicians is also essential to develop and maintain sustainable local expertise.

c) **Epidemiological surveillance**

Strengthening epidemiological surveillance is essential to monitor the circulation dynamics of major public health arboviruses, such as DENV, YFV, ZIKV, WNV, CHIKV, RVFV, and CCHFV, anticipate outbreaks, enable rapid response, and optimize limited resources.

Access to reliable and rapid diagnostic tools at the point of care is essential, given the nonspecific nature of arboviral symptoms [59–62]. This includes molecular and serological assays for detecting antigens or antibodies. Strengthening the diagnostic and clinical skills of healthcare providers will enhance early detection and case management [63].

Periodic seroprevalence surveys can establish population immunity baselines and inform targeting vaccination strategies [64,65]. For example, the WHO recommends a minimum% seroprevalence threshold to guide dengue vaccination campaigns due to the risk of severe disease upon reinfection [66].

d) **Multisectoral and International Collaboration**

The ecology of arboviruses involves complex interactions between viruses, vectors, reservoirs, and environmental factors [67–70]. Addressing this complexity requires a One Health (OH) approach, integrating the efforts of human health professionals, veterinarians, entomologists, and environmental scientists [71–76]. Although there is institutional engagement in Guinea to promote the One Health approach, with the establishment of a coordination platform at both central and decentralized levels down to the community level, collaboration between different sectors and disciplines remains lacking. The effective implementation of the One Health approach requires coordinated actions, encompassing the planning and execution of arbovirus surveillance, outbreak response, and vector control activities [73,77,78], as isolated interventions often lead to ineffective responses.

Effective arbovirus control also necessitates regional and international cooperation. The transboundary nature of these diseases calls for shared surveillance data, joint response protocols, and harmonized prevention strategies. The COVID-19 pandemic underscored the importance of global solidarity in epidemic preparedness and response [13,79,80]. Guinea would benefit from international support for capacity building, resource mobilization, and technical assistance [13]. Participating in regional training workshops and research initiatives can also enhance local competencies and promote the adoption of best practices [57,81].

## Conclusions

This study highlights the circulation of several arboviruses in Guinea across human, vector animal populations. The observed diversity underscores the urgent need for strengthen epidemiological and entomological surveillance to better understand transmission dynamics and predict epidemic risks.

Improving access to diagnostics, enhancing healthcare training, and engaging communities are essential components of an effective response strategy. Moreover, adopting an integrated, multidisciplinary, and collaborative approach-both nationally and internationally- will help to reduce the burden of arboviral diseases and improve Guinea’s preparedness against future epidemics.

## Supporting information

S1 Table Evidence of molecular or serological detection of arboviruses in Guinea according to different host populations and vectors. BATV: Batai virus; BHAV: Bhanja virus; BUNV: Bunyamwera virus; CCHFV: Crimean-Congo hemorrhagic fever virus; CHIKV: Chikungunya virus; DENV: Dengue virus; FORV: Forecariah virus; KIV: Kindia virus; KOLEV: Kolente virus; MOSV: Mossuril virus; MPOV: M’Poko virus; ONNV: O’nyong-nyong virus; RVFV: Rift Valley fever virus); SABV: Saboya virus; SINV: Sindbis virus; TAHV: Tahyna virus; USUV: Usutu virus; WNV: West Nile virus; YFV: Yellow fever virus; ZIKV: Zika virus.
(DOCX)

## References

[1] Chala B, Hamde F. Emerging and re-emerging vector-borne infectious diseases and the challenges for control: A review. Front Public Health. 2021;9:715759. doi: 10.3389/fpubh.2021.715759 34676194 PMC8524040

[2] Girard M, Nelson CB, Picot V, Gubler DJ. Arboviruses: A global public health threat. Vaccine. 2020;38(24):3989–94. doi: 10.1016/j.vaccine.2020.04.011 32336601 PMC7180381

[3] World Health Organization. Global arbovirus initiative: preparing for the next pandemic by tackling mosquito-borne viruses with epidemic and pandemic potential. Geneva: World Health Organization; 2024 24. https://iris.who.int/handle/10665/376630

[4] World Health Organization. Dengue and severe dengue. 2024 [cited 31 Aug 2024]. https://www.who.int/news-room/fact-sheets/detail/dengue-and-severe-dengue

[5] Centers for Disease Control and Prevention. Unveiling the Burden of Dengue in Africa | Blogs | CDC. 2015 [cited 18 Apr 2024]. https://blogs.cdc.gov/publichealthmatters/2015/07/unveiling-the-burden-of-dengue-in-africa/

[6] Bangoura ST, Keita A-K, Diaby M, Sidibé S, Le-Marcis F, Camara SC, et al. Arbovirus Epidemics as Global Health Imperative, Africa, 2023. Emerg Infect Dis. 2025;31(2):1–8. doi: 10.3201/eid3102.240754 39983695 PMC11845133

[7] Bangoura ST, Sidibé S, Kaba L, Mbaye A, Hounmenou CG, Diallo A, et al. Seroprevalence of seven arboviruses of public health importance in sub-Saharan Africa: A systematic review and meta-analysis. BMJ Glob Health. 2024;9(10):e016589. doi: 10.1136/bmjgh-2024-016589 39486798 PMC11529691

[8] World Health Organization, Diseases UBSP for R and T in T. Surveillance and control of arboviral diseases in the WHO African region: assessment of country capacities. Geneva: World Health Organization; 2022 36. https://iris.who.int/handle/10665/364827

[9] National Agency for Health Security. Guide to multisectoral management of priority zoonotic diseases in Guinea. Guinée; 2019 58. https://thecompassforsbc.org/project-examples/guide-de-gestion-multisectorielle-des-zoonoses-prioritaires-en-guinee-2018-2019

[10] Agence Nationale de Sécurité Sanitaire. Plan d’action national de la sécurité sanitaire de Guinée 2024-2028. Guinea, Conakry: Ministry of Health; 2024 84.

[11] Abbasi E. Global expansion of Aedes mosquitoes and their role in the transboundary spread of emerging arboviral diseases: A comprehensive review. IJID One Health. 2025;6:100058. doi: 10.1016/j.ijidoh.2025.100058

[12] Findlater A, Bogoch II. Human mobility and the global spread of infectious diseases: A focus on air travel. Trends Parasitol. 2018;34(9):772–83. doi: 10.1016/j.pt.2018.07.004 30049602 PMC7106444

[13] Longbottom J, Walekhwa AW, Mwingira V, Kijanga O, Mramba F, Lord JS. Aedes albopictus invasion across Africa: the time is now for cross-country collaboration and control. Lancet Glob Health. 2023;11(4):e623–8. doi: 10.1016/S2214-109X(23)00046-3 36841255

[14] Ministère de l’environnement, des eaux et forêts. Stratégie nationale sur le changement climatique - Guinée. Republique de Guinée: Ministère de l’environnement, des eaux et forêts; 2019 134.

[15] Ateutchia Ngouanet S, Wanji S, Yadouleton A, Demanou M, Djouaka R, Nanfack-Minkeu F. Factors enhancing the transmission of mosquito-borne arboviruses in Africa. Virusdisease. 2022;33(4):477–88. doi: 10.1007/s13337-022-00795-7 36278029 PMC9579656

[16] Li Y, Kamara F, Zhou G, Puthiyakunnon S, Li C, Liu Y, et al. Urbanization increases Aedes albopictus larval habitats and accelerates mosquito development and survivorship. PLoS Negl Trop Dis. 2014;8(11):e3301. doi: 10.1371/journal.pntd.0003301 25393814 PMC4230920

[17] Centre d’Observation, de Surveillance et d’Information Environnementales (COSIE). Rapport sur l’état de l’environnement en Guinée. Guinea: Ministère de l’Environnement, des Eaux et Forêts; 2012 317. https://www.stat-guinee.org/images/Documents/Publications/SSN/meef/rap%202012_COSIE.pdf

[18] National Statistical Institute. Statistical Yearbook 2021, Guinea. Guinea: National Statistical Institute; 2022 289. https://www.stat-guinee.org/images/Documents/Publications/INS/annuelles/annuaire/Annuaire_Statistique_2021_vf.pdf

[19] National Statistical Institute. General Population and Housing Recensement (RGPH-3 Guinea): household characteristics. Republic of Guinea. Conakry, Guinea; 2014.

[20] National Malaria Control Programme. National Malaria Strategic Plan 2018-2023, Guinea. Guinea, Conakry: Ministry of Health; 2019. https://portail.sante.gov.gn/wp-content/uploads/2023/02/GN-_PSN-2018-2023_Consolid%C3%A9-Atlantic-_26112019-revu-AC-PNLP-1.pdf

[21] World Health Organization. Regional Office for Africa. Integrated Disease Surveillance and Response Technical Guidelines: Booklet Three. World Health Organization. Regional Office for Africa; 2019. Report No.: WHO/AF/WHE/CPI/03, 2019. https://apps.who.int/iris/handle/10665/312362

[22] World Health Organization. WHO Immunization - Yellow Fever vaccination coverage. In: Immunization Data. [cited 26 Mar 2025]. https://immunizationdata.who.int/global/wiise-detail-page

[23] World Health Organization. A global strategy to Eliminate Yellow fever Epidemics 2017–2026. Geneva: World Health Organization; 2018. Licence: CC BY-NCSA 3.0 IGO. 2018 [cited 27 July 2023]. https://apps.who.int/iris/handle/10665/272408

[24] World Health Organization, United Nations Children’s Fund. Estimates of National Immunization Coverage (WUENIC). 2021. https://www.who.int/teams/immunization-vaccines-and-biologicals/immunization-analysis-and-insights/global-monitoring/immunization-coverage/who-unicef-estimates-of-national-immunization-coverage

[25] Programme Elargi de Vaccination. Pan Pluri-Annuel Complet (PPAC) PEV – GUINEE 2011-2015. République de Guinée: Ministère de la santé et de l’hygiène publique; 2011. https://apip.gov.gn/ficheprojet/1609763556.pdf

[26] Chopra H, Patel N, Sethi Y, Emran TB. Resurgence of yellow fever in Africa in 2022: A glance on protective measures. Int J Surg. 2023;109(2):112–4. doi: 10.1097/JS9.0000000000000117 36799819 PMC10389304

[27] Nwaiwu AU, Musekiwa A, Tamuzi JL, Sambala EZ, Nyasulu PS. The incidence and mortality of yellow fever in Africa: A systematic review and meta-analysis. BMC Infect Dis. 2021;21(1):1089. doi: 10.1186/s12879-021-06728-x 34688249 PMC8536483

[28] World Health Organization. A global strategy to eliminate yellow fever epidemics (EYE) 2017–2026. World Health Organization; 2018. https://apps.who.int/iris/handle/10665/272408

[29] World Health Organization. Disease Outbreak News; Yellow fever in East, West, and Central Africa. 2023 [cited 11 Nov 2025]. https://www.who.int/emergencies/disease-outbreak-news/item/2022-DON431

[30] Garske T, Van Kerkhove MD, Yactayo S, Ronveaux O, Lewis RF, Staples JE, et al. Yellow Fever in Africa: estimating the burden of disease and impact of mass vaccination from outbreak and serological data. PLoS Med. 2014;11(5):e1001638. doi: 10.1371/journal.pmed.1001638 24800812 PMC4011853

[31] Musso D, Ko AI, Baud D. Zika Virus Infection - After the Pandemic. N Engl J Med. 2019;381(15):1444–57. doi: 10.1056/NEJMra1808246 31597021

[32] World Health Organization. Zika epidemiology update - May 2024. 2024 [cited 5 Nov 2025]. https://www.who.int/publications/m/item/zika-epidemiology-update-may-2024

[33] Kraemer MUG, Brady OJ, Watts A, German M, Hay SI, Khan K, et al. Zika virus transmission in Angola and the potential for further spread to other African settings. Trans R Soc Trop Med Hyg. 2017;111(11):527–9. doi: 10.1093/trstmh/try001 29394415 PMC5914323

[34] Lourenço J, de Lourdes Monteiro M, Valdez T, Monteiro Rodrigues J, Pybus O, Rodrigues Faria N. Epidemiology of the Zika Virus Outbreak in the Cabo Verde Islands, West Africa. PLoS Curr. 2018;10:ecurrents.outbreaks.19433b1e4d007451c691f138e1e67e8c. doi: 10.1371/currents.outbreaks.19433b1e4d007451c691f138e1e67e8c 29637009 PMC5866102

[35] Sassetti M, Zé-Zé L, Franco J, Cunha J da, Gomes A, Tomé A, et al. First case of confirmed congenital Zika syndrome in continental Africa. Trans R Soc Trop Med Hyg. 2018;112(10):458–62. doi: 10.1093/trstmh/try074 30053235

[36] Sow A, Loucoubar C, Diallo D, Faye O, Ndiaye Y, Senghor CS, et al. Concurrent malaria and arbovirus infections in Kedougou, southeastern Senegal. Malar J. 2016;15:47. doi: 10.1186/s12936-016-1100-5 26821709 PMC4730666

[37] Oderinde BS, Mora-Cárdenas E, Carletti T, Baba MM, Marcello A. Prevalence of locally undetected acute infections of Flaviviruses in North-Eastern Nigeria. Virus Res. 2020;286:198060. doi: 10.1016/j.virusres.2020.198060 32561377

[38] Osoro E, Inwani I, Mugo C, Hunsperger E, Verani JR, Omballa V, et al. Prevalence of microcephaly and Zika virus infection in a pregnancy cohort in Kenya, 2017-2019. BMC Med. 2022;20(1):291. doi: 10.1186/s12916-022-02498-8 36100910 PMC9470235

[39] Shaibu JO, Okwuraiwe AP, Jakkari A, Dennis A, Akinyemi KO, Li J, et al. Sero-molecular Prevalence of Zika Virus among Pregnant Women Attending Some Public Hospitals in Lagos State, Nigeria. EJMED. 2021;3(5):77–82. doi: 10.24018/ejmed.2021.3.5.1075

[40] Boiro I, Lomonossov NN, Malenko GV, Balde C, Bah A. Forécariah virus, a new representative of the Bhanja antigenic group, isolated in the Republic of Guinea. Bull Soc Pathol Exot Filiales. 1986;79(2):183–6. 3731364

[41] Centers for Disease Control and Prevention. ArboCat Virus: Forecariah (FORV). [cited 22 Nov 2025]. https://wwwn.cdc.gov/arbocat//VirusDetails.aspx?ID=150&SID=8&utm_source=chatgpt.com

[42] Kartashov MY, Gladysheva AV, Naidenova EV, Zakharov KS, Shvalov АN, Krivosheina EI, et al. Molecular and genetic characteristics of the multicomponent flavi-like Kindia tick virus (Flaviviridae) found in ixodes ticks on the territory of the Republic of Guinea. Vopr Virusol. 2023;67(6):487–95. doi: 10.36233/0507-4088-145 37264838

[43] Kartashov MY, Krivosheina EI, Naidenova EV, Zakharov KS, Shvalov AN, Boumbaly S, et al. Simultaneous Detection and Genome Analysis of the Kindia Tick Virus in Cattle and Rhipicephalus Ticks in the Republic of Guinea. Vector Borne Zoonotic Dis. 2025;25(7):470–5. doi: 10.1089/vbz.2024.0056 40405773

[44] Centers for Disease Control and Prevention. ArboCat Virus: Kindia (KINV). [cited 22 Nov 2025]. https://wwwn.cdc.gov/arbocat/VirusDetails.aspx?ID=239&SID=8&utm_source=chatgpt.com

[45] Tsishevskaya AA, Alkhireenko DA, Bayandin RB, Kartashov MY, Ternovoi VA, Gladysheva AV. Untranslated regions of a segmented kindia tick virus genome are highly conserved and contain multiple regulatory elements for viral replication. Microorganisms. 2024;12(2):239. doi: 10.3390/microorganisms12020239 38399643 PMC10893285

[46] Ghedin E, Rogers MB, Widen SG, Guzman H, Travassos da Rosa APA, Wood TG, et al. Kolente virus, a rhabdovirus species isolated from ticks and bats in the Republic of Guinea. J Gen Virol. 2013;94(Pt 12):2609–15. doi: 10.1099/vir.0.055939-0 24062532 PMC3836499

[47] Bangoura ST, Hounmenou CG, Sidibé S, Camara SC, Mbaye A, Olive M-M, et al. Exploratory analysis of the knowledge, attitudes and perceptions of healthcare workers about arboviruses in the context of surveillance in the Republic of Guinea. PLoS Negl Trop Dis. 2023;17(12):e0011814. doi: 10.1371/journal.pntd.0011814 38048341 PMC10721174

[48] Díaz C, Torres Y, Cruz AM de la, Alvarez AM, Piquero ME, Valero A, et al. An inter-sector participatory strategy in Cuba using an ecosystem approach to prevent dengue transmission at the local level. Cad Saude Publica. 2009;25 Suppl 1:S59-70. doi: 10.1590/s0102-311x2009001300006 19287867

[49] King KF, Kolopack P, Merritt MW, Lavery JV. Community engagement and the human infrastructure of global health research. BMC Med Ethics. 2014;15:84. doi: 10.1186/1472-6939-15-84 25495054 PMC4290104

[50] Caprara A, Lima JWDO, Peixoto ACR, Motta CMV, Nobre JMS, Sommerfeld J, et al. Entomological impact and social participation in dengue control: A cluster randomized trial in Fortaleza, Brazil. Trans R Soc Trop Med Hyg. 2015;109(2):99–105. doi: 10.1093/trstmh/tru187 25604760 PMC4299523

[51] Echaubard P, Thy C, Sokha S, Srun S, Nieto-Sanchez C, Grietens KP, et al. Fostering social innovation and building adaptive capacity for dengue control in Cambodia: A case study. Infect Dis Poverty. 2020;9(1):126. doi: 10.1186/s40249-020-00734-y 32883345 PMC7469325

[52] Morales-Perez A, Nava-Aguilera E, Legorreta-Soberanis J, Paredes-Solís S, Balanzar-Martínez A, Serrano-de Los Santos FR, et al. Which green way: description of the intervention for mobilising against Aedes aegypti under difficult security conditions in southern Mexico. BMC Public Health. 2017;17(Suppl 1):398. doi: 10.1186/s12889-017-4300-1 28699562 PMC5506570

[53] Sánchez L, Pérez D, Alfonso L, Castro M, Sánchez LM, Van der Stuyft P, et al. A community education strategy to promote participation in dengue prevention in Cuba. Rev Panam Salud Publica. 2008;24(1):61–9. doi: 10.1590/s1020-49892008000700008 18764996

[54] Tapia-Conyer R, Méndez-Galván J, Burciaga-Zúñiga P. Community participation in the prevention and control of dengue: Thepatio limpiostrategy in Mexico. Paediatr Int Child Health. 2012;32(sup1):10–3. doi: 10.1179/2046904712z.00000000047 22668443 PMC3381439

[55] Amoa-Bosompem M, Kobayashi D, Murota K, Faizah AN, Itokawa K, Fujita R, et al. Entomological Assessment of the Status and Risk of Mosquito-borne Arboviral Transmission in Ghana. Viruses. 2020;12(2):147. doi: 10.3390/v12020147 32012771 PMC7077231

[56] Balthazar TD, Maia DA, Oliveira AA, Marques WA, Bastos AQ, Vilela ML, et al. Entomological surveillance of mosquitoes (Diptera: Culicidae), vectors of arboviruses, in an ecotourism park in Cachoeiras de Macacu, state of Rio de Janeiro-RJ, Brazil. PLoS One. 2021;16(12):e0261244. doi: 10.1371/journal.pone.0261244 34941927 PMC8699951

[57] Dadzie SK, Akorli J, Coulibaly MB, Ahadji-Dabla KM, Baber I, Bobanga T, et al. Building the capacity of West African countries in Aedes surveillance: Inaugural meeting of the West African Aedes Surveillance Network (WAASuN). Parasit Vectors. 2022;15(1):381. doi: 10.1186/s13071-022-05507-0 36271451 PMC9585720

[58] World Health Organization. Global vector control response 2017–2030. Geneva: World Health Organization. Licence: CC BY-NC-SA 3.0 IGO. 2017 [cited 31 Jan 2024]. https://www.who.int/publications-detail-redirect/9789241512978

[59] Jones RT, Tytheridge SJ, Smith SJ, Levine RS, Hodges MH, Ansumana R, et al. The threat of vector-borne diseases in sierra leone. Am J Trop Med Hyg. 2023;109(1):10–21. doi: 10.4269/ajtmh.22-0495 37277107 PMC10323989

[60] Ramphal Y, Tegally H, San JE, Reichmuth ML, Hofstra M, Wilkinson E, et al. Understanding the Transmission dynamics of the chikungunya virus in Africa. Pathogens. 2024;13(7):605. doi: 10.3390/pathogens13070605 39057831 PMC11279734

[61] Taylor-Robinson AW. Harnessing artificial intelligence to enhance key surveillance and response measures for arbovirus disease outbreaks: The exemplar of Australia. Front Microbiol. 2023;14:1284838. doi: 10.3389/fmicb.2023.1284838 37954250 PMC10634219

[62] Varghese J, De Silva I, Millar DS. Latest advances in arbovirus diagnostics. Microorganisms. 2023;11(5):1159. doi: 10.3390/microorganisms11051159 37317133 PMC10223626

[63] Chang S-C. Raising clinical awareness for better dengue fever outbreak control. J Formos Med Assoc. 2015;114(11):1025–6. doi: 10.1016/j.jfma.2015.10.006 26585886

[64] Tambo E, Khayeka-Wandabwa C, Olalubi OA, Adedeji AA, Ngogang JY, Khater EI. Addressing knowledge gaps in molecular, sero-surveillance and monitoring approaches on Zika epidemics and other arbovirus co-infections: A structured review. Parasite Epidemiol Control. 2017;2(2):50–60. doi: 10.1016/j.parepi.2017.01.001 29774281 PMC5952677

[65] Whelan MG, Ware H, Ranka H, Kenny S, Shaikh S, Roell Y, et al. ArboTracker: A multipathogen dashboard and data platform for arbovirus seroprevalence studies. Lancet Infect Dis. 2024;24(11):e670–1. doi: 10.1016/S1473-3099(24)00585-1 39270692

[66] World Health Organization = Organisation mondiale de la Santé. WHO position paper on dengue vaccines – May 2024 = Note de synthèse: position de l’OMS sur les vaccins contre la dengue – mai 2024. Weekly Epidemiological Record = Relevé épidémiologique hebdomadaire. 2024;99: 203–224. https://iris.who.int/handle/10665/376642

[67] Kraemer MUG, Sinka ME, Duda KA, Mylne AQN, Shearer FM, Barker CM, et al. The global distribution of the arbovirus vectors Aedes aegypti and Ae. albopictus. Elife. 2015;4:e08347. doi: 10.7554/eLife.08347 26126267 PMC4493616

[68] McIntyre KM, Setzkorn C, Hepworth PJ, Morand S, Morse AP, Baylis M. Systematic assessment of the climate sensitivity of important human and domestic animals pathogens in Europe. Sci Rep. 2017;7(1):7134. doi: 10.1038/s41598-017-06948-9 28769039 PMC5541049

[69] Mordecai EA, Ryan SJ, Caldwell JM, Shah MM, LaBeaud AD. Climate change could shift disease burden from malaria to arboviruses in Africa. Lancet Planet Health. 2020;4(9):e416–23. doi: 10.1016/S2542-5196(20)30178-9 32918887 PMC7490804

[70] Poongavanan J, Lourenço J, Tsui JL-H, Colizza V, Ramphal Y, Baxter C, et al. Dengue virus importation risks in Africa: a modelling study. Lancet Planet Health. 2024;8(12):e1043–54. doi: 10.1016/S2542-5196(24)00272-9 39674194 PMC11649930

[71] Conrad PA, Meek LA, Dumit J. Operationalizing a One Health approach to global health challenges. Comp Immunol Microbiol Infect Dis. 2013;36(3):211–6. doi: 10.1016/j.cimid.2013.03.006 23711930

[72] Dente MG, Riccardo F, Nacca G, Ranghiasci A, Escadafal C, Gaayeb L, et al. Strengthening preparedness for arbovirus infections in mediterranean and black Sea Countries: A conceptual framework to assess integrated surveillance in the context of the one health strategy. Int J Environ Res Public Health. 2018;15(3):489. doi: 10.3390/ijerph15030489 29534445 PMC5877034

[73] Dente MG, Riccardo F, Bolici F, Colella NA, Jovanovic V, Drakulovic M, et al. Implementation of the One Health approach to fight arbovirus infections in the Mediterranean and Black Sea Region: Assessing integrated surveillance in Serbia, Tunisia and Georgia. Zoonoses Public Health. 2019;66(3):276–87. doi: 10.1111/zph.12562 30724030 PMC6850493

[74] Faburay B. The case for a “one health” approach to combating vector-borne diseases. Infect Ecol Epidemiol. 2015;5:28132. doi: 10.3402/iee.v5.28132 26027713 PMC4450247

[75] Fouque F, Gross K, Leung Z, Boutsika K. Introduction to a landscape analysis of multisectoral approaches for prevention and control of infectious and vector-borne diseases. J Infect Dis. 2020;222(Suppl 8):S695–700. doi: 10.1093/infdis/jiaa489 33119097 PMC7594243

[76] Herdiana H, Sari JFK, Whittaker M. Intersectoral collaboration for the prevention and control of vector borne diseases to support the implementation of a global strategy: A systematic review. PLoS One. 2018;13(10):e0204659. doi: 10.1371/journal.pone.0204659 30303996 PMC6179246

[77] World Health Organization. Multisectoral approach to the prevention and control of vector-borne diseases: a conceptual framework. Geneva: World Health Organization; 2020 208. https://iris.who.int/handle/10665/331861

[78] World Health Organization. Launch of the Global Arbovirus Initiative. 2022 [cited 13 Jan 2024]. https://www.who.int/news-room/events/detail/2022/03/31/default-calendar/global-arbovirus-initiative

[79] Jit M, Ananthakrishnan A, McKee M, Wouters OJ, Beutels P, Teerawattananon Y. Multi-country collaboration in responding to global infectious disease threats: Lessons for Europe from the COVID-19 pandemic. Lancet Reg Health Eur. 2021;9:100221. doi: 10.1016/j.lanepe.2021.100221 34642675 PMC8495250

[80] Okesanya OJ, Olatunji G, Manirambona E, Oluebube MM, Rasheed A-SA, Olaleke NO, et al. Synergistic fight against future pandemics: Lessons from previous pandemics. Infez Med. 2023;31(4):429–39. doi: 10.53854/liim-3104-2 38075409 PMC10705866

[81] Braack L, Wulandhari SA, Chanda E, Fouque F, Merle CS, Nwangwu U, et al. Developing African arbovirus networks and capacity strengthening in arbovirus surveillance and response: Findings from a virtual workshop. Parasit Vectors. 2023;16(1):129. doi: 10.1186/s13071-023-05748-7 37059998 PMC10103543

