# Supplementary material for: Arboviruses circulation in Guinea: Overview and perspectives for public health
Source: PLoS Negl Trop Dis. 2026 Jan 9;20(1):e0013904. doi: 10.1371/journal.pntd.0013904 (PMC12788658; doi:10.1371/journal.pntd.0013904)
Supplement: S1 Table — BATV: Batai virus; BHAV: Bhanja virus; BUNV: Bunyamwera virus; CCHFV: Crimean-Congo hemorrhagic fever virus; CHIKV: Chikungunya virus; DENV: Dengue virus; FORV: Forecariah virus; KIV: Kindia virus; KOLEV: Kolente virus; MOSV: Mossuril virus; MPOV: M’Poko virus; ONNV: O’nyong-nyong virus; RVFV: Rift Valley fever virus); SABV: Saboya virus; SINV: Sindbis virus; TAHV: Tahyna virus; USUV: Usutu virus; WNV: West Nile virus; YFV: Yellow fever virus; ZIKV: Zika virus. (DOCX) [file pntd.0013904.s001.docx]

S1 Table: List of studies conducted in Guinea on arboviruses in humans, vectors, and animals up to 2024

| **Population** | **Population type** | **Arboviruses** | **Period of studies** | **Sources** |
| --- | --- | --- | --- | --- |
| **Humans** | Patients with fever | DENV | 2016-2017; 2006 | [1,2] |
|  |  | YFV | 2016-2017; 2006 | [1,2] |
|  |  | ZIKV | 2018-2021; 2016-2017 | [1,3] |
|  |  | WNV | 2006 | [2] |
|  |  | CHIKV | *ND*; 2006 | [2,4] |
|  |  | TAHV | 2006 | [2] |
|  |  | BUNV | 1978-1991 | [5] |
|  |  | CCHFV | 2016-2017 | [1] |
|  | General population | CCHFV | 2016-2019; *ND* | [6,7] |
|  |  | WNV | *ND*; 2020-2023 | [7,8] |
|  |  | BATV | *ND* | [7] |
|  |  | BHAV | *ND* | [7] |
|  |  | DENV | *ND*; 2020-2023 | [7,8] |
|  |  | CHIKV | *ND* | [7] |
|  |  | SINV | *ND* | [7] |
|  |  | YFV | 2020-2023 | [8] |
| **Vectors** | Mosquitoes | DENV | 1978-1991 | [5] |
|  |  | MOSV | 1978-1991 | [5] |
|  |  | MPOV | 1978-1991 | [5] |
|  | Ticks | CCHFV | 1978-1991; 1978-1985; 2016-2019; 2022 | [5,6,9,10] |
|  |  | KOLEV | 1978-1991 | [5] |
|  |  | KIV | *ND*; 1978-1991; 2021; 1981-1983; 1978-1985 | [5,9,11–13] |
|  |  | FORV | *ND*; 1978-1991; 2021; 1981-1983; 1978-1985 | [5,9,13,14] |

S1 Table: List of studies conducted in Guinea on arboviruses in humans, vectors, and animals up to 2024 (continued)

| **Population** | **Population type** | **Arboviruses** | **Period of studies** | **Sources** |
| --- | --- | --- | --- | --- |
| **Animals** | Nonhuman primates | DENV | 1978-1991 | [5] |
|  |  | RVFV | 1978-1991 | [5] |
|  |  | CHIKV | 1978-1991 | [5] |
|  |  | CCHFV | 1978-1991 | [5] |
|  |  | BUNV | 1978-1991 | [5] |
|  |  | MOSV | 1978-1991 | [5] |
|  |  | MPOV | 1978-1991 | [5] |
|  | Bats | DENV | 2016-2019 | [15] |
|  |  | ZIKV | 2016-2019 | [15] |
|  |  | WNV | 2016-2019 | [15] |
|  |  | USUV | 2016-2019 | [15] |
|  |  | CHIKV | 2016-2019 | [15] |
|  |  | ONNV | 2016-2019 | [15] |
|  |  | KOLEV | 1978-1991 | [5] |
|  |  | SABV | 1978-1991 | [5] |
|  | Wild mammals | RVFV | 1978-1989 | [16] |
|  |  | SABV | 1978-1989 | [16] |
|  | Wild birds | SABV | 1978-1991 | [5] |
|  | farm animals | CCHFV | 2018-2022 | [17] |
|  |  | RVFV | 2018-2022 | [17] |

BATV: Batai virus; BHAV: Bhanja virus; BUNV: Bunyamwera virus; CCHFV: Crimean-Congo hemorrhagic fever virus; CHIKV: Chikungunya virus; DENV: Dengue virus; FORV: Forecariah virus; KIV: Kindia virus; KOLEV: Kolente virus; MOSV: Mossuril virus; MPOV: M’Poko virus; ONNV: O’nyong-nyong virus; RVFV: Rift Valley fever virus); SABV: Saboya virus; SINV: Sindbis virus; TAHV: Tahyna virus; USUV: Usutu virus; WNV: West Nile virus; YFV: Yellow fever virus; ZIKV: Zika virus; ND: Not defined.

1. Dedkov VG, Magassouba N, Stukolova OA, Savina VA, Camara J, Soropogui B, et al. Differential Laboratory Diagnosis of Acute Fever in Guinea: Preparedness for the Threat of Hemorrhagic Fevers. Int J Environ Res Public Health. 2021;18: 6022. doi:10.3390/ijerph18116022

2. Jentes ES, Robinson J, Johnson BW, Conde I, Sakouvougui Y, Iverson J, et al. Acute Arboviral Infections in Guinea, West Africa, 2006. Am J Trop Med Hyg. 2010;83: 388–394. doi:10.4269/ajtmh.2010.09-0688

3. Bayandin RB, Makenov MT, Boumbaly S, Stukolova OA, Gladysheva AV, Shipovalov AV, et al. The First Case of Zika Virus Disease in Guinea: Description, Virus Isolation, Sequencing, and Seroprevalence in Local Population. Viruses. 2023;15: 1620. doi:10.3390/v15081620

4. Ivanov AP, Ivanova OE, Lomonosov NN, Pozdnyakov SV, Konstantinov OK, Bah MA. Serological investigations of Chikungunya virus in the Republic of Guinea. Ann Soc Belg Med Trop. 1992;72: 73–74.

5. Butenko AM. Arbovirus circulation in the Republic of Guinea. Med Parazitol (Mosk). 1996; 40–45.

6. Naidenova EV, Zakharov KS, Kartashov MY, Agafonov DA, Senichkina AM, Magassouba N, et al. Prevalence of Crimean-Congo hemorrhagic fever virus in rural areas of Guinea. Ticks Tick Borne Dis. 2020;11: 101475. doi:10.1016/j.ttbdis.2020.101475

7. Naidenova E, My K, Zakharov K, Shevtsova A, Diallo M, Nourdine I, et al. Study of the prevalence of antibodies to some arboviruses in the population of the Republic of Guinea. Vopr Virusol . 2021;66: 346–353. doi:10.36233/0507-4088-74

8. Krivosheina EI, И КЕ, Kartashov MY, Ю КМ, Naidenova EV, В НЕ, et al. Identification of specific IgG class antibodies to certain flaviviruses in the population of the Republic of Guinea. Russian Journal of Infection and Immunity. 2024;14: 381–386. doi:10.15789/2220-7619-IOS-15081

9. Konstantinov OK. Ticks of the Ixodidae family as reservoir of arboviruses in the Republic of Guinea. II. Arboviruses. Rev Elev Med Vet Pays Trop. 1990;43: 15–22.

10. Diallo MG, Boiro A, Balde TAL, Naydenova E, Bah BSS, Boumbaly S, et al. Circulation of Crimean-Congo haemorrhagic fever virus in ticks in Middle Guinea-Republic of Guinea. World Journal of Advanced Research and Reviews. 2023;20: 1087–1092. doi:10.30574/wjarr.2023.20.2.2306

11. Boiro I, Lomonossov NN, Alexin AF, Bah A, Balde C. Isolation of a new orbivirus Kindia (Palyam group) from ticks Amblyomma variegatum in the Republic of Guinea. Bull Soc Pathol Exot Filiales. 1986;79: 187–190.

12. Kartashov MY, Gladysheva AV, Naidenova EV, Zakharov KS, Shvalov АN, Krivosheina EI, et al. Molecular and genetic characteristics of the multicomponent flavi-like Kindia tick virus (Flaviviridae) found in ixodes ticks on the territory of the Republic of Guinea. Vopr Virusol. 2023;67: 487–495. doi:10.36233/0507-4088-145

13. Konstaninov OK, Butenko AM, Bashkirtsev VN, Libev MB, Marinina VP. Ticks of the family Ixodidae in Guinea and the isolation of arboviruses from them. II. Results of virologic and serologic research. Med Parazitol (Mosk). 1989; 9–13.

14. Boiro I, Lomonossov NN, Malenko GV, Balde C, Bah A. Forécariah virus, a new representative of the Bhanja antigenic group, isolated in the Republic of Guinea. Bull Soc Pathol Exot Filiales. 1986;79: 183–186.

15. Raulino R, Thaurignac G, Keita AK, Esteban A, Goumou S, Diallo R, et al. Seroprevalence of IgG Antibodies Against Multiple Arboviruses in Bats from Cameroon, Guinea, and the Democratic Republic of Congo. Vector Borne Zoonotic Dis. 2022;22: 252–262. doi:10.1089/vbz.2021.0076

16. Konstantinov OK, Diallo SM, Inapogi AP, Ba A, Kamara SK. The mammals of Guinea as reservoirs and carriers of arboviruses. Med Parazitol (Mosk). 2006; 34–39.

17. Naidenova EV, Kartashov MY, Shevtsova AP, Shipovalov AV, Kabanov AS, Boldyrev ND, et al. Identification of the Farm Animals Immune to Pathogens of Zoonotic Infectious Diseases in the Republic of Guinea. Problems of Particularly Dangerous Infections. 2022;0: 101–106. doi:10.21055/0370-1069-2022-2-101-106
